# METTL3 Regulates the Inflammatory Response in CPB2 Toxin-Exposed IPEC-J2 Cells through the TLR2/NF-κB Signaling Pathway

**DOI:** 10.3390/ijms232415833

**Published:** 2022-12-13

**Authors:** Juanli Zhang, Jiaojiao Yang, Xiaoli Gao, Xiaoyu Huang, Ruirui Luo, Qiaoli Yang, Zunqiang Yan, Pengfei Wang, Wei Wang, Kaihui Xie, Jie Li, Bo Zhang, Shuangbao Gun

**Affiliations:** 1 College of Animal Science and Technology, Gansu Agricultural University, Lanzhou 730070, China; 2 Gansu Academy of Agricultural Sciences (CAAS), Lanzhou 730070, China; 3 College of Animal Science and Technology, Northwest A&F University, Xi’an 712100, China; 4 Gansu Research Center for Swine Production Engineering and Technology, Lanzhou 730070, China

**Keywords:** METTL3, CPB2 toxin, IPEC-J2, inflammatory response, TLR2/NF-κB

## Abstract

*Clostridium perfringens* beta2 (CPB2) toxin is one of the main pathogenic toxins produced by *Clostridium perfringens*, which causes intestinal diseases in animals and humans. The N6-methyladenosine (m6A) modification is the most common reversible modification in eukaryotic disease processes. Methyltransferase-like 3 (METTL3) regulates immunity and inflammatory responses induced by the bacterial infections in animals. However, METTL3′s involvement in CPB2-treated intestinal porcine epithelial cell line-J2 (IPEC-J2) remains unclear. In the current study, we used methylated RNA immunoprecipitation-quantitative polymerase chain reaction, Western blotting and immunofluorescence assay to determine the role of METTL3 in CPB2-exposed IPEC-J2 cells. The findings revealed that m6A and METTL3 levels were increased in CPB2 treated IPEC-J2 cells. Functionally, METTL3 overexpression promoted the release of inflammatory factors, increased cytotoxicity, decreased cell viability and disrupted tight junctions between cells, while the knockdown of METTL3 reversed these results. Furthermore, METTL3 was involved in the inflammatory response of IPEC-J2 cells by activating the TLR2/NF-κB signaling pathway through regulating TLR2 m6A levels. In conclusion, METTL3 overexpression triggered the TLR2/NF-κB signaling pathway and promoted CPB2-induced inflammatory responses in IPEC-J2 cells. These findings may provide a new strategy for the prevention and treatment of diarrhea caused by *Clostridium perfringens*.

## 1. Introduction

In newborn animals such as pigs, sheep, cattle, dogs, cats, and fish, *Clostridium perfringens* type C causes abrupt mortality and hemorrhagic necrotizing enteritis [1,2,3]. Shockingly, 80–100% of infected piglets die [4,5], resulting in enormous economic losses for the worldwide pig industry. The pathogen produces α and β toxins [6,7], with *Clostridium perfringens* beta toxin being the key pathogenic toxin. The *Clostridium perfringens* beta1 toxin affects the small intestinal vascular endothelial cells, causing intestinal vascular injury, bleeding and tissue necrosis [8]. In addition, it activates the tachykinin NK1 receptor by promoting interleukin-1-beta (IL-1β) and tumor necrosis alpha (TNF-α), inducing inflammation in the animal body [9,10]. On the other hand, *Clostridium perfringens* beta2 (CPB2) toxin is a strong lethal toxin and cytotoxic encoded by the CPB2 gene in infected piglets with *Clostridium perfringens* type C and necrotizing enteritis [11]. Additionally, the expression of the CPB2 gene is 91.8% higher in piglets with enteritis, compared to 11.1% in healthy piglets [12]. According to the findings of several research studies, intestinal porcine epithelial cell line-J2 (IPEC-J2) reduces its activity when exposed to CPB2, which increases permeability, decreases the sexpression of the tight junction protein, and triggers an inflammatory response, i.e., releases inflammatory cytokines [13,14].

Currently, approximately 150 types of chemical modification in the RNA have been found, with RNA methylation modification accounting for more than 60% [15] and N6-methyladenosine (m6A) being the most extensively modified in rRNA, mRNA, tRNA, microRNA, small nuclear nucleic acid (snRNA), and long non-coding RNAs precursor [16,17]. Three key proteins regulate the m6A biological process, namely writers, erasers and readers. The writer complexes, including methyltransferase-like 14 (METTL14) [18], Wilms ’tumor 1-associating protein (WTAP) [19], and methyltransferase-like 3 (METTL3) [20], are essential in biological processes, helping to catalyze mRNA and non-coding RNA methylation. The erasers include alkylation repair homolog protein 5 (ALKHB5) [21] and fat mass and obesity-associated (FTO) protein [22], both key proteins in the demethylation of m6A in RNA. The readers contain a YT521B homology (YTH) domain [23], which recognizes the motif modified by m6A and activates the downstream regulatory pathways such as mRNA degradation and miRNA processing [24,25].

The Toll-like receptors (TLRs) are a type of pattern recognizing receptor, play a vital role in innate immunity and the inflammation response via the NF-kappa B (NF-κB)-mediated signaling pathway [26,27]. MyD88 and the Toll/IL-1R domain of MyD88, a unique adaptor protein used in the myeloid differentiation primary response, activate NF-κB to control the production of proinflammatory cytokine genes in response to Gram-positive bacteria, as well as damage-related pattern molecules (DAMPs) [28,29]. The transcription factor NF-κB coordinates the innate and adaptive immune responses, and thus is a key factor in the inflammatory responses and cell cycle [30]. In most cell types, NF-κB is composed of a p65/p50 heterodimer, which, once activated, releases the p65 subunit from the NF-κB inhibitorα (IκBα) and activates the transcription of target genes [31,32,33,34]. One of the most important components of the methyltransferase complex, which catalysis the production of m6A, is METTL3 [35]. The depletion of the METTL3 gene in lipopolysaccharide (LPS)-induced human dental pulp cells reduces the phosphorylation of IKK alpha/beta (IKKα/β), NF-κB (p65) and inhibitor of kappa B alpha (IκBα) molecules in the NF-κB signaling pathway, as well as it reduces p38 mitogen-activated protein kinase, JNK molecules and extracellular signal-regulated kinase (ERK) in the mitogen-activated protein kinase (MAPK) pathway in human dental pulp cells (HDPCs) [36]. Additionally, METTL3 knockdown suppresses the TLR signaling after LPS stimulation in macrophages [37]. However, the mechanism of METTL3 during the inflammatory responses induced by CPB2 in IPEC-J2 cells is still unknown. Therefore, the current study determines the role of METTL3 in IEPC-J2 cells stimulated to produce inflammatory responses by CPB2. This study’s results may shed light on the IPEC-J2 cell’s inflammatory responses when exposed to CPB2.

## 2. Results

### 2.1. Total m6A Contents and METTL3 Expression Levels Are Upregulated in IPEC-J2 Cells

**Figure 1:**
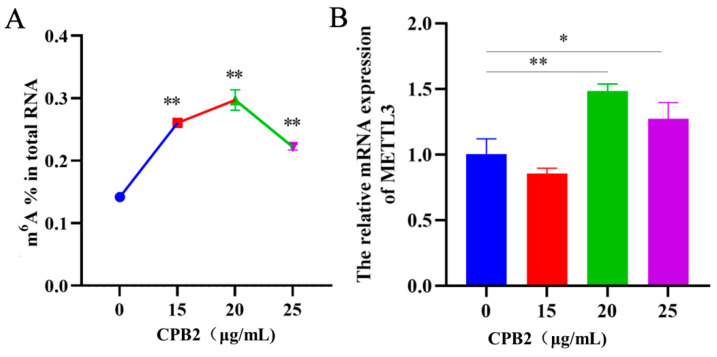
Total m6A content and METTL3 expression level in CPB2 toxin-treated cells. (**A**) The m6A content in IPEC-J2 cells after 24 h of treatment with 0, 15, 20, and 25 μg/mL CPB2 toxin. (**B**) METTL3 mRNA expression in IPEC-J2 cells treated for 24 h with 0, 15, 20, or 25 μg/mL CPB2. All data are based on three separate trials and presented as mean ± SD. Statistically significant change from CPB2 toxin concentrations of 0 μg/mL (* *p* < 0.05; ** *p* < 0.01).

To investigate the effect of CPB2 toxin stimulation on RNA m6A modification in IPEC-J cells, the overall m6A methylation level was measured. The results showed that the overall level of m6A methylation was significantly higher in IPEC-J2 cells treated with 15, 20 and 25 μg/mL CPB2 toxin for 24 h compared to the control (0 μg/mL), peaking at 20 μg/mL toxin concentration treatment (Figure 1A, *p* < 0.01). Next, qPCR was performed to analyze the mRNA levels of m6A methyltransferase (METTL3). The expression of METTL3 peaked at 20 μg/mL CPB2 toxin treatment for 24 h (Figure 1B, *p* < 0.01). Thus, we used 20 μg/mL CPB2 toxin-treated IPEC-J2 cells for 24 h as our research subjects in the following investigations.

### 2.2. METTL3 Promotes the Overall Level of m6A Methylation in IPEC-J2 Cells

**Figure 2:**
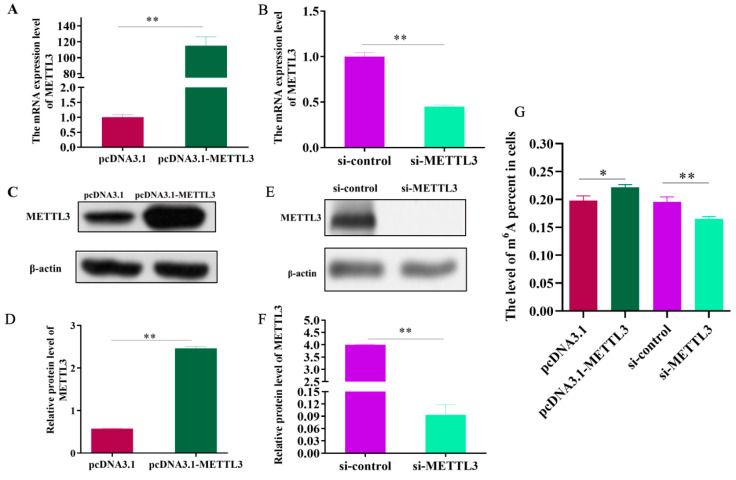
The effect of METTL3 on m6A methylation levels in IPEC-J2 cells. The expressions of METTL3 mRNAs (**A**,**B**) and Western blotting (**C**–**F**) after METTL3 knockdown or overexpression in IPEC-J2 cells. (**G**) IPEC-J2 cell m6A content following METTL3 overexpression and knockdown. All data are based on three separate trials presented as mean ± SD. ** *p* < 0.01 and * *p* < 0.05, respectively.

To explore the function of METTL3, we knocked down or overexpressed METTL3 in IPEC-J2 cells. As shown in the figure (Figure 2A–F, *p* < 0.01), the mRNA and protein levels of METTL3 were significantly increased after METTL3 overexpression, while the mRNA and protein levels were significantly decreased after METTL3 knockdown. The subsequent functional research on METTL3 used these cells. The overexpression of METTL3 considerably raised the m6A level in IPEC-J2 cells, whereas the knockdown of METTL3 dramatically lowered m6A levels (Figure 2E, *p* < 0.05, *p* < 0.01). Based on the results above, METTL3 positively regulates the levels of methylation in IPEC-J2 cells.

### 2.3. Overexpressing METTL3 in IPEC-J2 Cells Induces CPB2-Induced Inflammation

**Figure 3:**
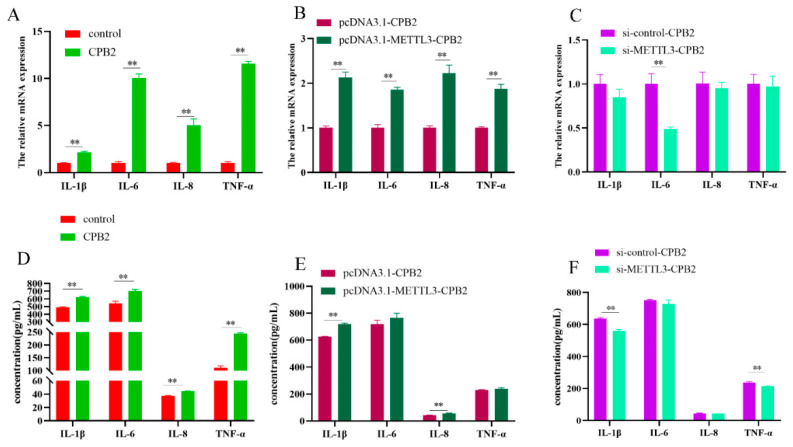
Effect of METTL3 on pro-inflammatory factor expression in IPEC-J2 cells exposed to CPB2 for 24 h. Expression levels of pro-inflammatory factors IL-1β, IL-6, IL-8, and TNF-α mRNA (**A**–**C**) and protein (**D**–**F**) in IPEC-J2 cells. All data are based on three separate trials and presented as mean ± SD. ** *p* < 0.01.

Following CPB2 treatment of IPEC-J2 cells, RT-PCR showed that *IL-1β*, *IL-6*, *IL-8* and *TNF-α* expressions were significantly elevated in IPEC-J2 cells, and the overexpression of METTL3 further promoted the release of pro-inflammatory factors *IL-1β*, *IL-6*, *IL-8* and *TNF-α*, while the knockdown of METTL3 significantly reduced *IL-6* expression (Figure 3A–C, *p* < 0.01). In addition, ELISA assays verified that CPB2 treatment significantly increased the release of pro-inflammatory factors *IL-1β*, *IL-6*, *IL-8* and *TNF-α* from IPEC-J2 cells at the protein level (Figure 3D, *p* < 0.01). The overexpression of METTL3 further promoted the release of pro-inflammatory factors in IPEC-J2 cells, while the knockdown of METTL3 inhibited the expression of pro-inflammatory factors (Figure 3E,F). These results revealed that METTL3 overexpression promoted the inflammatory response in CPB2-treated IPEC-J2 cells, while METTL3 knockdown inhibited the inflammatory response.

### 2.4. Overexpression of METTL3 Decreases Cell Viability of CPB2-Exposed IPEC-J2

**Figure 4:**
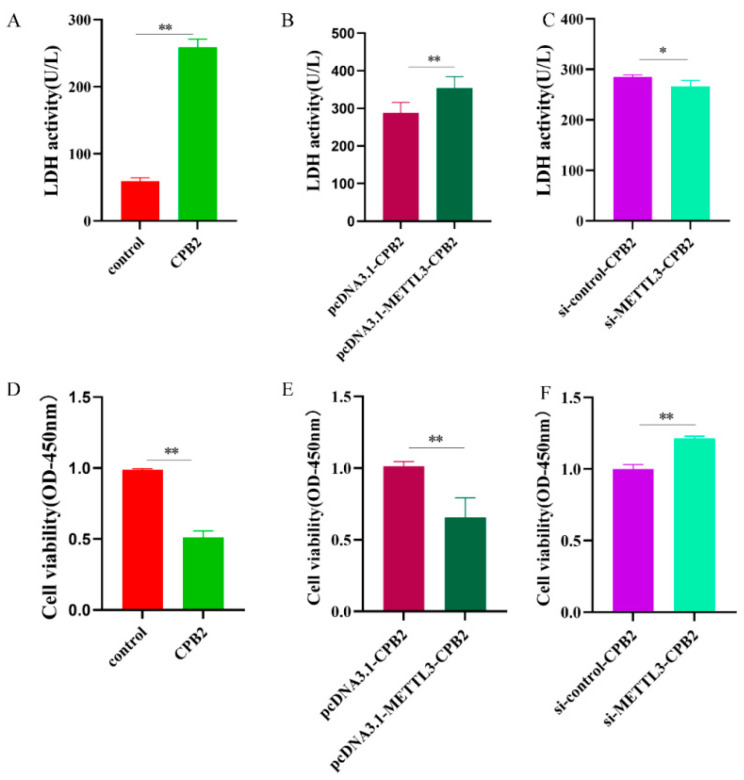
The impacts of METTL3 overexpression or knockdown on cytotoxicity and cell survival in CPB2-exposed IPEC-J2 cells. (**A**–**C**) The activity of LDH in IPEC-J2 cells and the results represent the absorbance at 450 nm. (**D**–**F**) IPEC-J2 cell viability as measured by the CCK-8 test. All data are based on three separate trials and presented as mean ± SD. ** *p* < 0.01 and * *p* < 0.05, respectively.

The IPEC-J2 cells were used to evaluate METTL3 for toxicity and cellular activity. The LDH assay showed an increase in cell cytotoxicity in the CPB2 group (Figure 4A, *p* < 0.01), and the overexpression of METTL3 enhanced LDH activity (Figure 4B, *p* < 0.01), whereas METTL3 knockdown decreased LDH activity (Figure 4C, *p* < 0.05). The CPB2 group cell viability was considerably lower than that of the control (Figure 4D, *p* < 0.01), and cell viability in the pcDNA3.1-METTL3-CPB2 group was also significantly lower than that in pcDNA3.1-CPB2 group (Figure 4E, *p* < 0.01), as demonstrated by the CCK8 assay. The si-METTL3-CPB2 group had considerably greater cell viability than si-control-CPB2 (*p* < 0.01), as shown in Figure 4F. These findings showed that CPB2 toxin was hazardous to IPEC-J2 cells and reduced their viability. METTL3 enhanced cell toxicity and decreased cell viability in IPEC-J2 cells.

### 2.5. METTL3 Knockdown Promotes Expression of Tight Junction-Related Proteins

**Figure 5:**
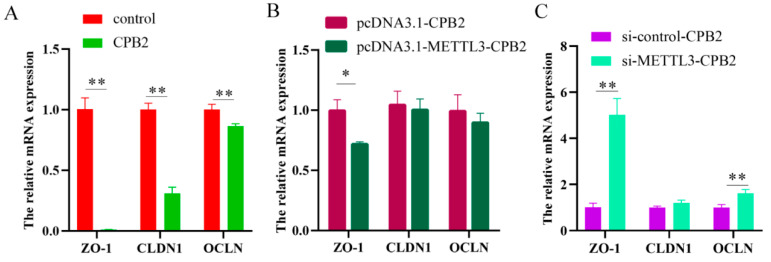
Tight junction protein mRNA expression. mRNA expression of tight junction protein (*ZO-1*, *CLDN1*, *OCLN*) in the control and CPB2 groups (**A**), pcDNA3.1-CPB2 and pcDNA3.1-METTL3-CPB2 groups (**B**), and si-control-CPB2 and si-METTL3-CPB2 groups (**C**). All data are based on three separate trials and presented as mean ± SD. ** *p* < 0.01 and * *p* < 0.05, respectively.

**Figure 6:**
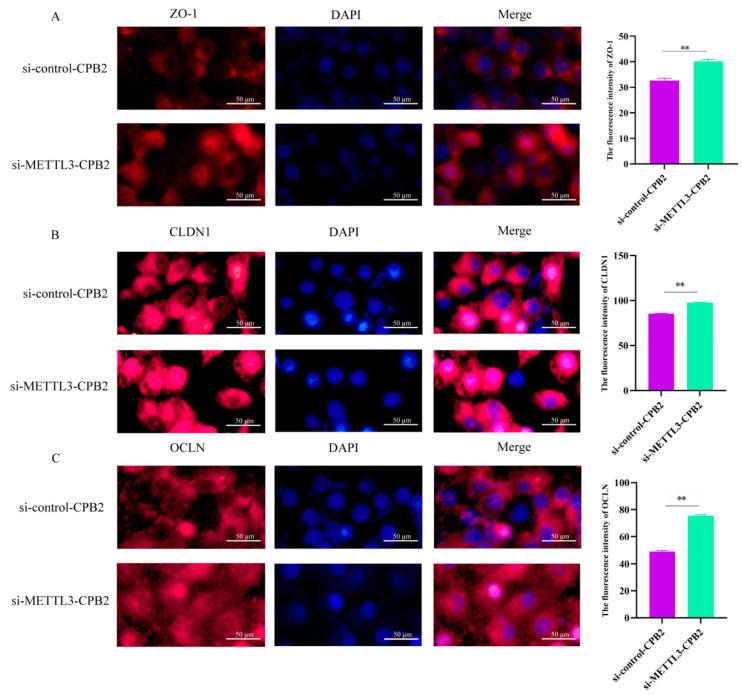
Tight junction protein expression after METTL3 knockdown after CPB2 toxin treatment. (**A**–**C**) CLDN1, ZO-1, and OCLN immunofluorescence intensity in the si-control-CPB2 and si-METTL3-CPB2 groups. As a secondary antibody, IgG/Cy3 was utilized. The intensity of the red fluorescence represents the level of protein expression. The blue colour represents cell nucleus. Three bar graphs depict group annotations. The scale bar is 50 μm. All of the data are based on three separate experiments and are given as mean ± SD. ** *p* < 0.01.

To determine the impact of METTL3 overexpression and knockdown on CPB2 toxin-treated IPEC-J2 cells, the tight junction-related genes’ (*CLDN1*, *ZO-1*, and *OCLN*) expression was evaluated. After treatment with CPB2 toxin, there was a statistically significant decrease in these mRNA expression levels (Figure 5A, *p* < 0.01). In particular, METTL3 overexpression in CPB2-induced IPEC-J2 cells reduced ZO-1 expression considerably (Figure 5B, *p* < 0.05), while METTL3 knockdown substantially elevated OCLN and ZO-1 expression (Figure 5C, *p* < 0.01). Immunofluorescence further showed that the fluorescence intensity of ZO-1, CLDN1 and OCLN proteins was significantly increased in CPB2-induced IPEC-J2 cells after METTL3 knockdown (Figure 6, *p* < 0.01).

### 2.6. Overexpression of METTL3 in CPB2-Induced IPEC-J2 Cells Triggers Inflammation through TLR2/NF-κB

**Figure 7:**
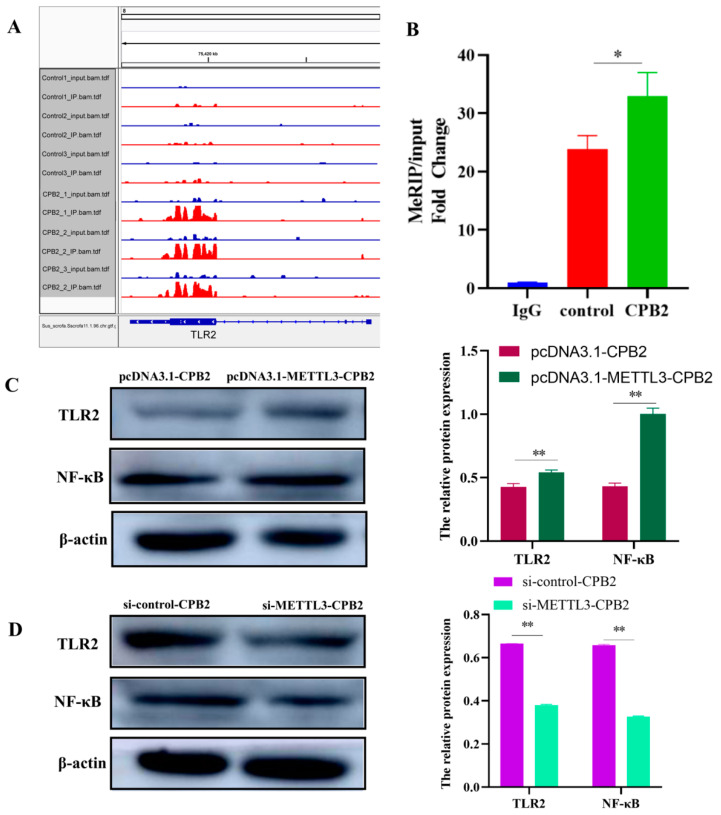
TLR2/NF-B pathway effects of METTL3 overexpression and knockdown in CPB2-exposed IPEC-J2 cells. (**A**) TLR2 m6A peak distribution in the control and CPB2 groups using the Integrative Genomics Viewer (IGV) program. (**B**) MeRIP-qPCR analysis of TLR2 m6A expression in IPEC-J2 cells exposed to CPB2. (**C**,**D**) TLR2 and NF-κB protein expression in CPB2-exposed cells following METTL3 overexpression and knockdown. Figure 3 shows the group annotations. All data are based on three separate trials and presented as mean ± SD. ** *p* < 0.01 and * *p* < 0.05.

**Figure 8:**
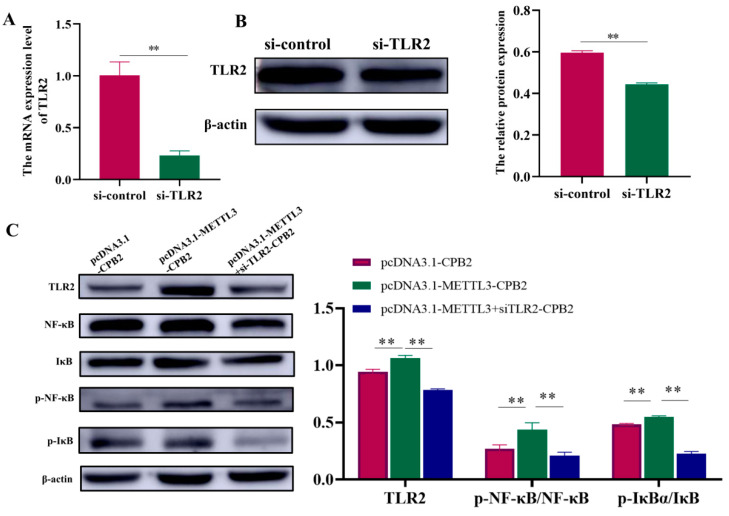
TLR2/NF-κB signaling pathway was stimulated by METTL3 by controlling TLR2 expression. TLR2 transfection effectiveness was measured using (**A**) RT-qPCR and (**B**) Western blot in IPEC-J2 cells. (**C**) TLR2, NF-κB, p-NF-κB, p-IκB, and IκB protein expression. All data are based on three separate trials and presented as mean ± SD. ** *p* < 0.01.

**Figure 9:**
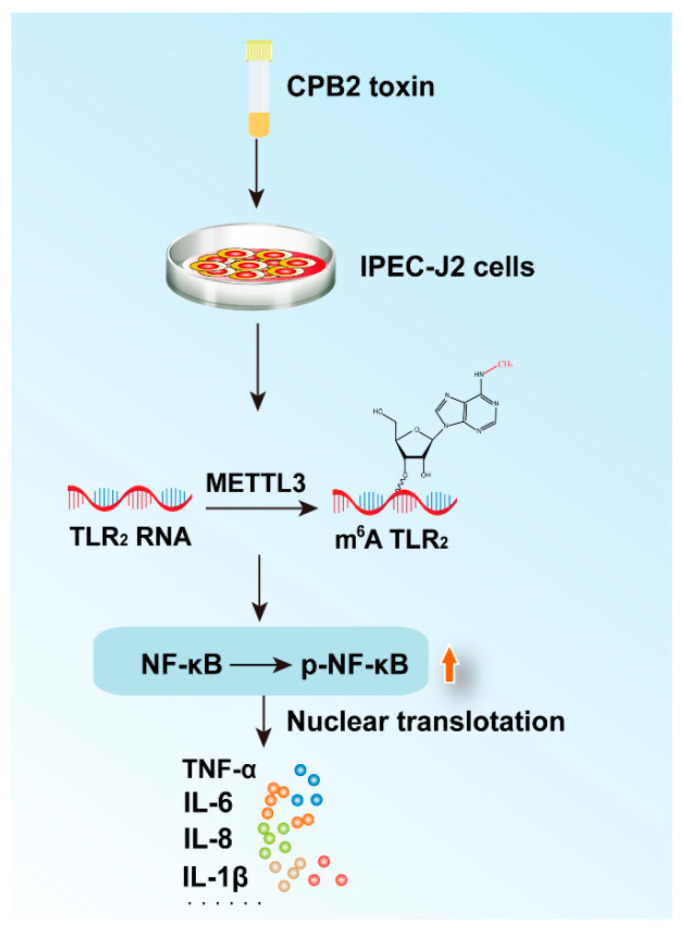
A schematic depiction showing METTL3 controlling inflammatory responses in CPB2-exposed IPEC-J2 cells through the TLR2/NF-κB pathway. TLR2 was activated by METTL3 through m6A methylation, and TLR2 in turn activated NF-κB, which was then phosphorylated into the nucleus, promoting the release of inflammatory cytokines. Red arrows indicate elevated levels of NF-κB protein phosphorylation.

The m6A-seq of the control and CPB2-treated cells revealed that the m6A peak and *TLR2* gene expression were significantly increased by the CPB2 toxin (Supplementary Table S1). Figure 7A shows the results of an analysis of the TLR2 m6A peak using the integrative Genomics Viewer software (IGV, version 2.8.13). This analysis confirmed that the CPB2 group upregulated the m6A peak in the IP library. The MeRIP-qPCR results show the significant increase in TLR2 m6A methylation level (Figure 7B, *p* < 0.05). Furthermore, TLR2 and NF-κB protein expression was enhanced after METTL3 overexpression in CPB2-induced IPEC-J2 cells (Figure 7C, *p* < 0.01) and was significantly decreased after *METTL3* knockdown (Figure 7D, *p* < 0.01). TLR2 was knocked down to confirm the regulatory link between the two proteins, i.e., METTL3 and TLR2 (Figure 8A,B, *p* < 0.01). TLR2, NF-κB, phosphorylated NF-κB (p-NF-κB), phosphorylated IκB (P-IκB), and inhibitor Kappa B (IκB) proteins were all found to be more highly expressed in the pcDNA3.1-METTL3-CPB2 group than in the pcDNA3.1-CPB2 (Figure 8C, *p* < 0.01). When pcDNA3.1-METTL3 and si-TLR2 were co-transfected in CPB2-induced IPEC-J2 cells, the expression of both factors was considerably reduced (Figure 8C, *p* < 0.01). For the pcDNA3.1-METTL3-CPB2 group, the phosphorylation ratio of p-NF-κB/NF-κB to p-IκB/IκB was greater than in the pcDNA3.1–METTL3 + SiTLR2-CPB2 group (Figure 8C, *p* < 0.01). METTL3 overexpression thereby regulated TLR2 and led to a series of reactions (Figure 9).

## 3. Discussion

The m6A modification is the RNA methylation modification of the nitrogen atom of adenine at the sixth position in a specific RNA region, while METTL3, also known as MT-A70, belongs to a large conserved methyltransferase family. The m6A methyltransferase complex comprises at least five “writer” proteins, such as METTL3, METTL14, WTAP, RBM15 and VIRMA. METTL3 plays a core catalytic role [38]. It was reported that METTL3 regulates inflammation and autoimmune balance [36,39]. In this work, CPB2-exposed IPEC-J2 cells showed substantially higher METTL3 expression and m6A content. This is in keeping with prior research, which found that LPS-stimulated periodontitis cells [36], microglia cells [40] and an IL-1β-stimulated ATDC5 chondroprogenitor cell line dramatically enhance METTL3 expression and m6A levels [41]. These findings suggested that METTL3 is involved in the inflammatory response in cells stimulated by different inflammatory factors through m6A modification.

METTL3 is a key factor in inflammatory responses induced by different diseases, where it promotes inflammation in different cells. For example, METTL3 promotes LPS-mediated inflammation and exerts anti-LCFA absorption activity in vitro [42]. Moreover, METTL3 knockdown in LPS-stimulated periodontitis cells showed a reduced level of expression of the inflammatory cytokines, i.e., IL-6 and IL-8 [41]. In addition, METTL3 knockdown in LPS-stimulated periodontitis cells reduces IL-6 and IL-8 production [36]. METLL3 overexpression boosted the release of these cytokines, whereas METLL3 knockdown inhibited the production of these cytokines. Preosteoblast MC3T3-E1 cells treated with LPS produce more pro-inflammatory cytokines when METTL3 is depleted [43]. METTL3 also suppresses macrophage-like cells’ inflammatory activities through the NF-κB pathway [35]. The results from this study show that METTL3 plays a critical role in controlling inflammation.

METTL3 regulates diverse signaling pathways in different diseases. The TLR2/NF-κB signaling pathway is now known to have a significant role in a wide range of diseases [44,45,46,47], including the inflammation of the intestinal tract [48,49]. There are various signal transduction pathways, including NF-κB signaling, which stimulates pro-inflammatory cytokine release and cyclooxygenase-2 enzymes [50], in response to microbial invasion [33,34,51]. In rats with 5-fluorouracil-induced colitis, the TLR2/MyD88/NF-κB pathway is activated to speed up inflammation, whereas patchouli alcohol (PA) slows down colitis through TLR2/MyD88/NF-κB pathway inhibition [50]. TLR2 and NF-κB were also upregulated by METTL3 overexpression, in contrast to METTL3 knockdown. It was also observed that the phosphorylation ratio of p-NF-κB/NF-κB and p-IκB/IκB was significantly increased in the pcDNA3.1-METTL3-CPB2 group, and significantly reduced in the pcDNA3.1-METTL3 + si-TLR2-CPB2 co-transfection group. In addition, the TLR2 m6A peak was elevated considerably. By changing the methylation of TLR2 to turn on the TLR2 gene, activating the NF-B signaling cascade, and increasing the release of pro-inflammatory cytokines, METTL3 increased the inflammatory response in IPEC-J2 cells that had been exposed to CPB2.

The tight junction protein is the most important barrier component constituting the epithelial cells, regulating intestinal barrier permeability [52]. Intestinal epithelial barrier dysfunction and increased permeability are the most common causes of intestinal infections [53]. The impact of METTL3 on CPB2-exposed IPEC-J2 cells was also investigated in this work. LDH is a very stable oxidoreductase that may be utilized to measure tissue cell damage and toxicity after cell injury [54,55]. In this study, METTL3 was shown to increase cell toxicity. After METTL3 overexpression, CCK8 assay demonstrated a reduction in cell viability. The knockdown of METTL3, on the other hand, improved cell survival. Furthermore, following METTL3 knockdown, OCLN, ZO-1, and CLDN1 expression was considerably enhanced, as indicated by the immunofluorescence experiment. In summary, in IPEC-J2 cells exposed to CPB2, METTL3 enhanced inflammatory response and intestinal mucosal injury.

As mentioned above, increased METTL3 expression triggered the TLR2/NF-κB signaling pathway, resulting in an increase in cytokine production. In addition, METTL3 knockdown reduced cell membrane damage. These findings are crucial in analyzing the cell inflammatory response from the epitranscriptome of the CPB2-exposed IPEC-J2 cells, providing a molecular mechanism for promising new treatments against diarrhea caused by *Clostridium perfringens*.

## 4. Materials and Methods

### 4.1. Cell Culture

In the current study, the utilized porcine IPEC-J2 cells were purchased from the Beina Chuanglian Biotechnology Institute (Beijing, China). The cells were seeded at a density of 1 × 10^5^ cells/mL in 24-well plates and grew on HyClone’s Modified Eagle Medium (DMEM, HyClone, Logan, UT, USA) supplemented with 1% penicillin–streptomycin solution (Beyotime, Shanghai, China) and 10% fetal bovine serum. Plates with 5% CO_2_ at 37 °C were incubated until 70% confluence.

### 4.2. CPB2 Treatment and Total m6A Measurement

The CPB2 toxin was obtained in the same manner as reported earlier [13,14]. After seeding on 24-well plates, IPEC-J2 cells were treated with CPB2 toxin for 24 h at several doses (0, 15, 20, and 25 μg/mL, respectively). Then, the cells were harvested. We then proceeded to extract RNA from cells, and the m6A level was quantified using the Colorimetric EpiQuik kit (Epigentek, New York, NY, USA). Briefly, 200 ng of total extracted RNA was added to each well of a 96-well plate, using a high-performance RNA binding solution; additionally, the antibody was added to detect m6A. The detected m6A was quantified using a standard curve generated by determining the absorbance at 450 nm wavelength. The quantity of m6A was proportional to the optical density’s (OD) measured intensity.

### 4.3. METTL3 Overexpression, Knockdown Transfection

The pcDNA3.1 vector was used to construct the METTL3 overexpression vector, and was then transfected into IPEC-J2 cells to upregulate METLL3 (here defined as pcDNA3.1-METTL3). The negative control was the pcDNA3.1 vector (here defined as pcDNA3.1). In addition, the cells were transfected with siRNA targeted METTL3 or TLR2 (here defined as si-METTL3 and si-TLR2) through lipofectamine 2000 (Invitrogen, Carlsbad, CA, USA), while non-targeting siRNA was used as the control (here defined as si-control), all designed and synthesized by GenePharma (Shanghai, China). The sequences of si-METTL3 and si-TLR2 were 5′-GACGGAUCAUCAAUAAACATT-3′ and 5′-GCCCUUCCUACACACUUUATT-3′, respectively.

### 4.4. Detection of Cell Viability and Lactate Dehydrogenase (LDH)

IPEC-J2 cells were seeded in a 96-well plate (5 × 10^3^ cells per well) and incubated for 24 h. The cells were subsequently transfected with si-METTL3, si-control, pcDNA3.1, and pcDNA3.1-METTL3. Following 24 h of incubation, the underlined transfected cells were exposed to 20 μg/mL CPB2 toxin for 24 h. Then, the LDH activity was evaluated through the LDH cytotoxicity assay kit (Beyotime, Shanghai, China). Finally, the OD intensities of CCK8 and LDH were measured at 450 and 490 nm, respectively.

### 4.5. Detection of Enzyme-Linked Immunosorbent Assays (ELISA) for Cytokine Detection

IPEC-J2 cells were transfected with si-METTL3, pcDNA3.1-METTL3, and the corresponding negative controls (si-control and pcDNA3.1). The cells were subsequently treated with CPB2 (20 μg/mL) followed by ELISA (Shanghai Enzyme-linked Biotechnology Co., Ltd., Shanghai, China) assays to assess pro-inflammatory cytokine expression.

### 4.6. Quantitative Reverse-Transcription PCR (RT-qPCR)

Total RNA was extracted from IPEC-J2 cells (1 × 10^7^) using TransZol reagent (TransGen Biotech, Beijing, China). The RNA was reverse transcribed using the Evo M-MLV RT Kit for qPCR II (Accurate Biotechnology, Changsha, China) and SYBR^®^ Green Premix Pro Taq HS qPCR Kit as per the instructions. GAPDH and the 2^−ΔΔCt^ technique were used to normalize RNA expression and compute RNA levels [56]. In this work, GENEWIZ Biotechnology Co., Ltd. (Tianjin, China) developed and synthesized all of the primers in Supplementary Table S2.

### 4.7. Western Blotting

The cultured cells were lysed in RIPA lysis buffer (KeyGEN, Nanjing, China) and protein content was determined via BCA Protein Assay Kit (Beyotime, Shanghai, China). Protein loading buffer was combined with the total protein solution at a 4:1 ratio and heated in a boiling water bath for 10 min for protein denaturing. These proteins were further separated through SDS-PAGE, and were further transferred to a polyvinylidene difluoride (PVDF) membrane (Millipore, Bedford, USA). Furthermore, the membrane was blocked for 2 h at room temperature in 1 × Tris Buffered Saline with Tween^®^20 (TBST) buffer with 5% nonfat milk, and then incubated overnight at 4 °C with the primary antibodies: METTL3 (1:2000, ab195352, Cambridge, USA), TLR2 (1:500, 17236-1-AP, USA), NF-κB (1:500, bs-0465R, Beijing, China), p-NF-κB (1:150, bs-3485R, China), IκB (1:500, AF-5002, Jiangsu, China), p-IκBα (1:500, bs-5515R, Beijing, China) and β-actin (1:5000, bs-0061R, Beijing, China). Next, the membrane was treated with secondary antibody and incubated for 1 h at room temperature before being detected through an enhanced chemiluminescence luminescent fluid (ECL) from Solarbio (Beijing, China).

### 4.8. Immunofluorescence Assay

The cells were washed thrice using phosphate-buffered saline (PBS), followed by fixing in 4% paraformaldehyde for 20 min at room temperature. Next, normal goat serum (1:20) was used for the cells’ blockage for 30 min, treated with the primary antibodies Zonula occludens 1 (ZO-1, bs-1329R, Beijing, China), claudin 1 (CLDN1, bs-1008R), and occludin (OCLN, bs-1495R), all at a dilution of 1:300, and incubated overnight at 4 °C. The cells were rewashed (thricely, 10 min each wash) with PBS, followed by incubation for 1 h at 37 °C with the fluorescent secondary antibody (IgG/Cy3, bs-0309P-Cy3, 1:300). The cells were washed three times with PBS, stained for 5 min with 4’,6-diamidino-2-phenylindole (DAPI), and then rinsed again with PBS. The cells were subsequently examined using an Olympus fluorescent microscope (Olympus IX71, Olympus, Tokyo, Japan).

### 4.9. Methylated RNA Immunoprecipitation-Quantitative Polymerase Chain Reaction (MeRIP-qPCR)

The 300 µg total RNA was isolated from the CPB2 and control groups. The m6A MeRIP Kit (genseq Inc., Shanghai, China) was used to fragment the extracted RNA, enrich it with m6A and IgG antibodies, and purify it. Finally, using the reverse transcription kit (Accurate Biotechnology, Changsha, China), the RNA was reverse transcribed into cDNA. The RT-qPCR assay was performed as above.

### 4.10. Statistical Analysis

The GraphPad Prism 9.0 (GraphPad Software, San Diego, CA, USA) for the statistical analysis, while ImageJ V1.8.2 (Rawak Software Inc., Stuttgart, Germany) was employed to quantify the immunofluorescence data and Western blot. Moreover, for the two and multiple group comparisons, Unpaired Student’s *t*-test, one-way ANOVA with Tukey’s multiple comparison test were used. The mean ± standard deviation (SD) for each experiment was computed in triplicate. A statistically significant result was defined as *p* < 0.05.

## 5. Conclusions

In conclusion, we report that m6A and METTL3 expression levels are upregulated in CPB2-stimulated IPEC-J2 cells and that METTL3 knockdown reduces cell membrane damage and increases cell viability. Mechanistically, increased METTL3 expression triggered the TLR2/NF-κB signaling pathway and promoted inflammatory responses in IPEC-J2 cells. These findings are key in analyzing the cell inflammatory response from the epitranscriptome of the CPB2-exposed IPEC-J2 cells, which provide a promising molecular mechanism for new treatment strategies against piglet diarrhea caused by *Clostridium perfringens*.

## Data Availability

Data is contained within the article or Supplementary Material.

## Supplementary Materials

The following supporting information can be downloaded at: https://www.mdpi.com/article/10.3390/ijms232415833/s1.
